# Physiological determinants of residual cerebral arterial pulsatility on best medical treatment after TIA or minor stroke

**DOI:** 10.1177/0271678X20969984

**Published:** 2020-11-05

**Authors:** Alastair JS Webb, Amy Lawson, Linxin Li, Sara Mazzucco, Peter M Rothwell, for the Oxford Vascular Study Phenotyped Cohort

**Affiliations:** Wolfson Centre for Prevention of Stroke and Dementia, University of Oxford, Oxford, UK

**Keywords:** Arterial stiffness, aortic blood pressure, age, cerebral pulsatility, small vessel disease

## Abstract

Cerebral arterial pulsatility is strongly associated with cerebral small vessel disease and lacunar stroke yet its dependence on central versus local haemodynamic processes is unclear. In a population-based study of patients on best medical managment, 4–6 weeks after a TIA or non-disabling stroke, arterial stiffness and aortic systolic, diastolic and pulse pressures were measured (Sphygmocor). Middle cerebral artery peak and trough flow velocities and Gosling’s pulsatility index were measured by transcranial ultrasound. In 981 participants, aortic and cerebral pulsatility rose strongly with age in both sexes, but aortic diastolic pressure fell more with age in men whilst cerebral trough velocity fell more in women. There was no significant association between aortic systolic or diastolic blood pressure with cerebral peak or trough flow velocity but aortic pulse pressure explained 37% of the variance in cerebral arterial pulsatility, before adjustment, whilst 49% of the variance was explained by aortic pulse pressure, arterial stiffness, age, gender and cardiovascular risk factors. Furthermore, arterial stiffness partially mediated the relationship between aortic and cerebral pulsatility. Overall, absolute aortic pressures and cerebral blood flow velocity were poorly correlated but aortic and cerebral pulsatility were strongly related, suggesting a key role for transmission of aortic pulsatility to the brain.

## Introduction

Small vessel disease accounts for approximately 30% of strokes and 40% of dementia^1^ and is associated with acute lacunar stroke,^2^ progressive cognitive decline,^3^ late-onset refractory depression,^4^ functional impairment in daily living^5^ and increased mortality.^6^ White matter hyperintensities are highly prevalent in the population, affecting over half of people over the age of 65 and the majority of people over 85. However, even patients with advanced imaging changes can remain functionally independent,^7^ indicating a pre-clinical stage of the disease where intervention may prevent progression of SVD and resulting clinical morbidity. White matter hyperintensities are strongly associated with a history of hypertension and are particularly associated with markers of vascular aging, including aortic stiffness and pulsatility of blood flow in the aorta and the cerebral circulation.^8^

Identifying methods to reduce cerebral arterial pulsatility and associated clinical harms would be informed by characterising the physiological processes responsible for elevated pulsatility, understanding the role of pulsatility in leading to clinical harm and identifying potential treatment targets. Pulsatility of cerebral blood flow is associated with distal arterial resistance and increased aortic stiffness and pulsatility of aortic blood flow. However, it is unclear what the magnitude of the association between cerebral arterial pulsatility and aortic blood pressure is, whether the relationship between aortic pulsatility and cerebral blood flow velocity is consistent across demographic groups and whether targeting aortic pulsatility and arterial stiffness could reduce cerebral arterial pulsatility and small vessel disease.

Therefore, in a large population with recent TIA or minor stroke with optimised blood pressure control and best medical treatment, we compared the population distribution and strength of association between residual cerebral pulsatility and aortic haemodynamic measures.

## Methods

### Study population

Consecutive, consenting patients with TIA or minor stroke were recruited between September 2010 and September 2019, as part of the Oxford Vascular Study (OXVASC).^9,10^ Participants were recruited at the OXVASC daily emergency assessment clinic, following a referral after attendance at the Emergency Department or from primary care, usually within 24 hours. Patients were referred after transient neurological symptoms or symptoms consistent with a minor stroke, not requiring direct admission to hospital. The OXVASC population consists of >92,000 individuals registered with about 100 primary-care physicians in Oxfordshire, UK.^11^ All consenting patients underwent a standardised medical history and examination, ECG, blood tests and a stroke protocol MRI brain and contrast-enhanced MRA (or CT-brain and carotid Doppler ultrasound or CT-angiogram), an echocardiogram and 5 day ambulatory cardiac monitor. All patients were assessed by a study physician, and reviewed by the senior study neurologist (PMR) and are followed-up face-to-face at 1, 3, 6 and 12 months, and 2, 5 and 10 years. Medication is standardly prescribed according to guidelines, most commonly with dual antiplatelets (aspirin and clopidogrel), high dose statins (atorvastatin 40-80mg) and a combination of perindopril and indapamide, with the addition of amlodipine as required, to reach a target of <130/80, guided by home telemetric blood pressure monitoring in the majority of participants.

As part of the OXVASC Phenotyped Cohort, a routine prospective cardiovascular physiological assessment is performed at the 1 month follow-up visit.^9,10^ Participants were excluded if they were under 18 years, cognitively impaired (MMSE < 23 at the ascertainment or one month visits), pregnant, had autonomic failure, a recent myocardial infarction, unstable angina, heart failure (NYHA 3-4 or ejection fraction <40%) or untreated bilateral carotid stenosis (>70%). OXVASC is approved by the Oxfordshire Research Ethics Committee A (05/Q1604/70), and is carried out in accordance with the Declaration of Helsinki, as revised in 1983. Written, informed consent was obtained from all participants.

Physiological tests were performed at rest in a quiet, dimly-lit, temperature-controlled room (21–23°C). Applanation tonometry (Sphygmocor, AtCor Medical, Sydney, Australia) was used to measure carotid-femoral pulse wave velocity (aortic-PWV), aortic augmentation index and aortic systolic and diastolic blood pressure and pulse pressure (ao-SBP, ao-DBP, ao-PP).^17^ Where applanation tonometry was not possible due to bilateral carotid stenosis or technical failure, pulse wave analysis and arterial stiffness was determined with a vicorder. The distribution of PWV and aortic measures determined by vicorder were transformed to match the population distribution of measures derived from a Sphygmocor,^12^ estimated in a subset of the population in whom both tests were performed, with sensitivity analyses performed restricted to patients undergoing assessment of PWV by Sphygmocor alone. TCD (Doppler Box, Compumedics DWL, Singen, Germany) was performed with a 2 MHz probe at the temporal bone window on the same side as carotid applanation, where possible. The waveform envelope was acquired at 100 Hz simultaneously with ECG and blood pressure at 200 Hz (Finometer, Finapres Medical Systems, The Netherlands), via a Powerlab 8/30 with LabChart Pro software (ADInstruments, USA). The MCA was insonated at the site of peak velocity closest to 50 mm, or if this was not adequate, at the depth giving the optimal waveform, excluding vessels with velocity transitions and magnitude indicative of a focal MCA stenosis, determined by the sonographer. All waveforms were visually inspected and beats corrupted by artefact were excluded. Absolute peak, trough and mean velocities were calculated as the average of the remaining beats during a 15 second window, from the envelope of the spectrum. MCA pulsatility was calculated as Gosling’s pulsatility index (MCA-PI= (systolic CBFV-diastolic CBFV)/mean CBFV).

Associations between demographic or physiological indices were determined by general linear models for continuous variables, logistic regression for binary outcomes and ordinal regression for ordinal outcomes. Models were performed for univariate associations; adjusted for age and sex; age, sex and cardiovascular risk factors and for cardiovascular risk factors and interactions between age and sex and cardiovascular risk factors. Relationships between systemic haemodynamic measures and cerebral pulsatility were determined by general linear models, with mediation analysis to identify the potential pathway of effect. Mean values and associations were determined stratified by age (in quintiles) and sex, with formal assessment of interactions through general linear models.

Analyses were performed in R (packages *tidyverse*, *mediation, emmeans*), Matlab r2015 or Windows Excel.

## Results

Of 1013 eligible participants, 981 had either arterial stiffness or TCD performed. 849 patients had TCD recordings (excluding 104 with no bone windows and 60 participants due to machine failure, lack of availability or participant refusal), of whom 801 had higher quality recordings. 936 patients had arterial stiffness measures whilst 767 patients underwent both tests. Patients with either TCD or arterial stiffness measures were younger than those with neither, were more likely to be in heart failure and were more likely to be women (Supplemental Table 1).

A history of hypertension was associated with increased SBP, DBP and aortic PP, but was also associated with a lower PSV and EDV but a significantly raised PI, before and after adjustment for age and other cardiovascular risk factors (Supplemental Tables 2 and 3). A history of diabetes was associated with increased PSV and MCA-PI, but there was no difference in EDV. Although current smoking was weakly associated with cerebral pulsatility before adjustment, and a lower SBP and aortic pulse pressure after adjustment for confounders, there were no independent associations between smoking with cerebrovascular indices after adjustment for risk factors.

**Figure 1.**
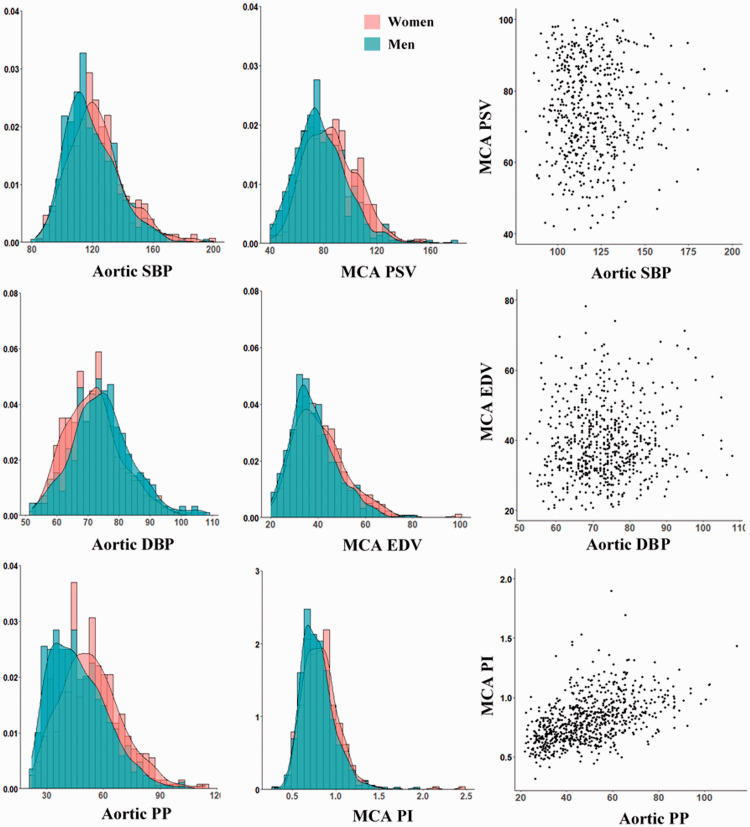
Distributions of cerebral physiological indices and aortic indices, and the relationship between them. Results are presented as histogram and kernel density plot, stratified by gender, and for the scatter plot between the cerebral and systemic measures. Results are presented for aortic systolic blood pressure (SBP), aortic diastolic blood pressure (DBP), aortic pulse pressure (PP), MCA peak systolic velocity (PSV), MCA end diastolic velocity (EDV) and MCA pulsatility index (PI).

**Figure 2.**
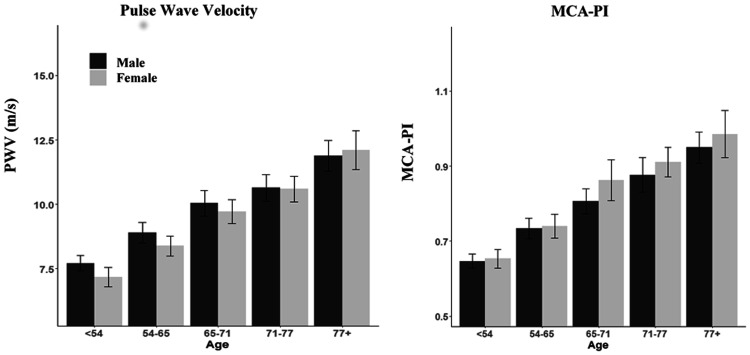
Age and sex distributions of aortic stiffness and cerebral arterial pulsatility. Y-axes are scaled to the 95% range for the whole population for each index. The population is presented in quintiles of age, divided into male and female. PWV = pulse wave velocity; MCA-PI = middle cerebral artery pulsatility index.

The age and sex distributions of aortic stiffness and aortic pulsatility were very similar to the distributions for MCA-PI, with age being particularly associated with increased PWV, aortic pulse pressure and MCA-pulsatility index (Figures 1 and 2). MCA-PI and aortic PP were both greater in women than men but this difference attenuated after adjustment for age, reflecting the difference in age distributions between the sexes. However, although MCA-PI increased with age similarly in both sexes, aortic-PP increased slightly more rapidly with age in women than men, whilst there was no significant difference in the increase in PWV between sexes (Figure 2, Table 1).

**Table 1.** Relationship between age and sex with key physiological indices.

	Model 1			Model 2		Adjusted	
	R^2^	Age (p)	Sex (p)	R^2^	Interact.	R^2^	Interact.
PWV (m/s)	0.31	<0.0001	0.21	0.31	0.041	0.35	0.08
HR (bpm)	0.01	0.83	0.002	0.01	0.24	0.03	0.36
SBP	0.08	<0.0001	0.58	0.10	<0.0001	0.10	<0.0001
DBP	0.05	0.018	<0.0001	0.07	<0.0001	0.07	0.001
Ao. SBP	0.10	<0.0001	0.005	0.11	<0.0001	0.12	0.001
Ao. DBP	0.05	<0.0001	<0.0001	0.06	0.002	0.05	0.004
Ao. PP	0.20	<0.0001	<0.0001	0.20	0.027	0.21	0.036
Ao AIX	0.28	<0.0001	<0.0001	0.28	0.026	0.30	0.03
MCA PSV	0.05	<0.0001	<0.0001	0.05	0.21	0.05	0.29
MCA EDV	0.23	<0.0001	<0.0001	0.23	0.022	0.24	0.04
MCA PI	0.29	<0.0001	0.02	0.29	0.25	0.31	0.22

Results of general linear models are presented for each index including age and sex (model 1), age and sex and the interaction between them (model 2) and for model 2 also adjusted for cardiovascular risk factors.

**Table 2.** Relationships between cerebral blood flow indices and systemic physiological indices.

	MCA PSV				MCA EDV				MCA PI			
	Univariate		Adjusted		Univariate		Adjusted		Univariate		Adjusted	
	R^2^	p	R^2^	p	R^2^	p	R^2^	p	R^2^	p	R^2^	p
PWV (m/s)	0.003	0.10	0.006	0.54	0.11	<0.001	0.007	0.10	0.22	<0.001	0.015	<0.001
Ao. AiX	0.005	0.03	0.005	0.068	0.003	0.069	−0.002	0.018	0.051	0	0.01	0.68
Ao. SBP	0	0.41	0.006	0.09	0.03	<0.001	0.001	0.42	0.097	<0.001	0.022	<0.001
Ao. DBP	0.006	0.027	0.006	0.076	0.006	0.03	0.001	0.28	0.07	<0.001	0.014	<0.001
Ao. PP	0.005	0.024	0.014	0.003	0.05	<0.001	0	0.05	0.23	<0.001	0.08	<0.001
Ao. MBP	0.001	0.20	0	0.87	0	0.28	−0.004	0.99	−0.002	0.74	0.024	0.34
MCA PSV	–	–	–	–	0.62	<0.001	0.53	<0.001	0.01	0.037	0.03	<0.001
MCA EDV	0.62	<0.001	0.66	<0.001	–	–	–	–	0.18	<0.001	0.08	<0.001
MCA PI	0.01	0.003	0.034	<0.001	0.18	<0.001	0.04	<0.001	–	–	–	–

Results are presented for general linear models as univariate analyses, and following adjustment for age, sex and cardiovascular risk factors. Results are presented for the adjusted R^2^ for the univariate association, and for the increase in R-squared with the addition of the physiological index to the model containing only age, gender and cardiovascular risk factors as predictors.

**Figure 3.**
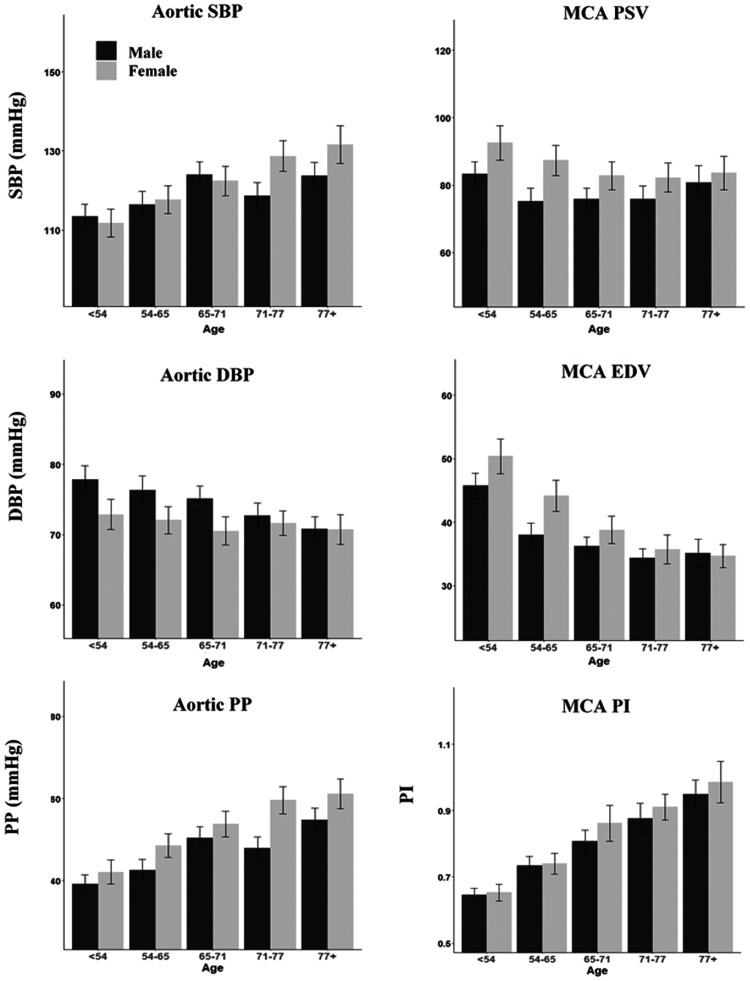
Age and sex distributions of cerebral and aortic physiological indices. Results are presented as the mean and confidence interval of each index, for each quintile of age split by sex. Results are presented for aortic systolic blood pressure (SBP), aortic diastolic blood pressure (DBP), aortic pulse pressure (PP), MCA peak systolic velocity (PSV), MCA end diastolic velocity (EDV) and MCA pulsatility index (PI). Y-axis limits are set to 95% range of recorded measures for each index.

In contrast to the relatively consistent age-sex distributions for systemic and central measures of pulsatility, absolute values of blood pressure and cerebral blood flow velocities differed. Aortic SBP increased with age in both sexes, but with a stronger relationship in women than men and a significant interaction between age and sex (Table 1). However, MCA-PSV fell with age in women but in men there was little change across age-groups (Figure 3). In contrast, EDV and DBP both fell with age but the fall in EDV was more pronounced in women (p = 0.0004), compared to a limited fall in aortic DBP in women versus men (Figure 3, Table 2).

There were at best weak associations between SBP with PSV and DBP with EDV, before and after adjustment for risk factors (Table 3, Supplemental Figure 1, Supplemental Table 4). In contrast, there was a strong association between aortic PP and MCA PI, which persisted after adjustment for age, sex and cardiovascular risk factors. There were similarly strong associations with aortic pulse wave velocity, with weaker associations with aortic augmentation index**.**

**Table 3.** Physiological determinants of cerebrovascular indices.

	MCA peak velocity						MCA trough velocity						MCA pulsatility index					
	Physiology		Adj Age/Sex		Adj CV RFs		Physiology		Adj Age/Sex		Adj CV RFs		Physiology		Adj Age/Sex		Adj CV RFs	
	β	p	β	p	β	p	β	p	β	p	β	p	β	p	β	p	β	p
*Model 1*	R^2^ =	0.01	R^2^ =	0.06	R^2^ =	0.07	R^2^ =	0.12	R^2^ =	0.24	R^2^ =	0.25	R^2^ =	0.37	R^2^ =	0.42	R^2^ =	0.43
PWV	−0.08	0.054	0.06	0.216	0.05	0.383	−0.27	<0.001	−0.05	0.301	−0.03	0.564	0.31	<0.001	0.18	<0.001	0.12	0.003
Aor-PP	0.13	0.007	0.14	0.007	0.15	0.006	−0.2	<0.001	−0.11	0.016	−0.08	0.111	0.51	<0.001	0.39	<0.001	0.37	<0.001
Aor-MBP	−0.07	0.129	−0.08	0.09	−0.07	0.149	0.12	0.005	0.06	0.151	0.05	0.22	−0.3	<0.001	−0.23	<0.001	−0.22	<0.001
																		
*Model 2*	R^2^ =	0	R^2^ =	0.06	R^2^ =	0.07	R^2^ =	0.12	R^2^ =	0.22	R^2^ =	0.23	R^2^ =	0.41	R^2^ =	0.46	R^2^ =	0.49
PWV	−0.03	0.47	0.13	0.021	0.12	0.043	−0.26	<0.001	−0.04	0.449	−0.01	0.825	0.38	<0.001	0.25	<0.001	0.19	<0.001
Aor-PP	0.06	0.253	0.08	0.208	0.07	0.241	−0.17	0.002	−0.1	0.069	−0.06	0.28	0.39	<0.001	0.31	<0.001	0.26	<0.001
Aor-MBP	−0.08	0.096	−0.09	0.105	−0.08	0.167	0.1	0.035	0.04	0.398	0.02	0.63	−0.32	<0.001	−0.25	<0.001	−0.23	<0.001
Aor-Aix	0.07	0.112	0.07	0.175	0.09	0.103	−0.02	0.589	0.02	0.601	0.04	0.393	0.13	<0.001	0.06	0.123	0.08	0.058
R-R int.	0	0.926	0.05	0.307	0.06	0.205	−0.09	0.039	−0.04	0.304	−0.05	0.28	0.13	<0.001	0.11	0.001	0.15	<0.001

Results of general linear models are presented for each cerebral blood flow index (PSV, EDV and PI) for model 1: including aortic pulse wave velocity (PWV), aortic pulse pressure (Aor-PP) and Aortic mean blood pressure (Aor-MBP); or for model 2: including all factors in model 1 plus Aortic augmentation index (Aor-Aix) and R-R interval, the inverse of heart rate. Both models are presented unadjusted for clinical characteristics, adjusted for age and sex and adjusted for age, sex and cardiovascular risk factors (diabetes, history of hypertension, current smoking, ever smoking).

In multivariate analyses of all haemodynamic aortic indices, there were only weak associations between absolute aortic pressure values and MCA PSV or EDV (Table 3), whether aortic BP was defined by aortic SBP and aortic DBP or aortic mean pressure (Supplemental Table 5). However, aortic pulse pressure was a significant predictor of absolute cerebral blood flow velocity after adjustment for age and sex and cardiovascular risk factors, albeit with loss of this association following adjustment for heart rate and augmentation index (Table 3).

In contrast, aortic pulse pressure was particularly strongly associated MCA-PI, predicting 37% of the variance in cerebral pulsatility index, with an inverse association with mean aortic BP. Addition of age, sex and cardiovascular risk factors only explained a further 6% of the variance in MCA-PI, with a small additional effect of adjusting for heart rate and augmentation index. The fully adjusted model explained nearly 50% of the variance in MCA-PI (Table 3). Similar effects were found for models defining aortic BP as aortic SBP and DBP rather than pulse pressure and MBP (Supplemental Table 2). Within the models, aortic PP remained the strongest predictor of MCA-PI, with a standardised beta coefficient of 0.37 exceeding even the standardised beta-coefficient for age (0.30).

In addition to the strong relationship between ao-PP and MCA-PI, PWV predicted an earlier arrival of the pulse wave at the MCA (MCA arrival time p < 0.0001), including after adjustment for age and sex (p = 0.003). Furthermore, aortic PWV partially mediated the relationship between aortic PP and MCA-PP, explaining 24% of the relationship between aoPP and MCA-PI (p < 0.0001), although this fell to 9% after adjustment for age and sex (p < 0.0001).

## Discussion

In a large population of patients with TIA or minor stroke, cerebral arterial pulsatility was strongly associated with aortic pulsatility and stiffness, with similar distributions by age, sex and cardiovascular risk factors. In contrast, there were minimal to weak relationships between absolute measures of aortic pressure (SBP or DBP) and absolute measures of cerebral blood flow velocity. There were additional independent associations between cerebral pulsatility with measures of arterial stiffness and heart rate, with small additional effects of age, sex or cardiovascular risk factors. Overall, nearly half of the variance in cerebral pulsatility across the population was explained, suggesting a strong dependence of cerebral pulsatility on aortic pulsatility and arterial stiffness.

Middle cerebral arterial pulsatility is strongly associated with cerebral small vessel disease,^8,13^ lacunar stroke^14,15^ and the risk of recurrent stroke, independently of age and sex, and independent of hypertension and diabetes. Cerebral pulsatility is a potential therapeutic target but its reduction requires a more detailed understanding of its determinants. It has previously been assumed to principally reflect changes in distal resistance in the brain due to in vitro modelling of its dependence on peripheral resistance and independence of myocardial contractility^16^ and clinical associations with cerebral small vessel disease,^17^ with the low resistance cerebral circulation having a high diastolic flow rate and more rounded waveforms. Our study demonstrates a strong dependence of MCA pulsatility on aortic pulse pressure and further demonstrates that this relationship is dependent upon arterial stiffening, particularly due to central stiffening (aortic PWV) with a likely contribution of increased wave reflection from peripheral vessels (augmentation index). Partial mediation of the aortic cerebral pulsatility relationship by arterial stiffness, and the association of aortic stiffness with early arrival time of the pulse, also implies that increased aortic stiffness increases transmission of the aortic waveform to the brain, increasing pulsatility in the middle cerebral artery.

Our study is the first and largest of its type to assess systemic cardiovascular physiology with cerebral haemodynamic function in the same population of high-risk individuals with TIA or minor stroke. As such, it provides the best description available of the comparative distribution of changes in arterial stiffness and cerebral physiological indices in this patient group, with similar distributions of aortic and cerebral indices with increasing age and by sex. Furthermore, it confirms associations with a history of hypertension and diabetes, but limited association with dyslipidaemia or a history of current or previous smoking, reaffirming the importance of effective blood pressure control in early to mid-life^18^ and consistent with recent reports of a strong dependence of arterial stiffness and associated vascular changes on mid-life hypertension.^18^

In contrast to the strong associations between aortic pulsatility with cerebral arterial pulsatilty, we demonstrated a comparatively weak association between absolute blood pressure indices and absolute measures of cerebral blood flow velocity. However, these patients had undergone significant blood pressure control that may have resulted in a short-term blood pressure reduction but persistent effects of previous hypertension on the cerebral vasculature, resulting in discordance between current aortic and cerebral values. This shifting of the ‘set-point’ of the relationship between absolute aortic pressures and cerebral blood flow velocities implies an important role of local determinants of absolute blood pressure and absolute blood flow velocity, such as cerebrovascular tone and stiffness. In contrast, the strong association between aortic and cerebral pulsatility, despite blood pressure lowering, implies that effects on systolic and diastolic pressures in an individual occur in parallel with a preserved relative difference, such that cerebral pulsatility still reflects enhanced transmission of the persistently pulsatile aortic waveform to the brain. Interventions to reduce cerebral pulsatility are therefore likely to require a reduction in aortic pulsatility,^19^ a reduction in the speed and magnitude of wave reflections or a reduction in arterial stiffness itself.^20^ The Eclipse study suggested that a vasodilating phosphodiesterase inhibitor, cilostazol, reduced cerebral arterial pulsatility,^21^ but did not also assess effects on aortic blood pressure and therefore could not determine the site of action. Similarly, the CAFÉ study^19^ suggested a greater effect of vasodilating antihypertesive medications on aortic blood pressure than vasoconstricting treatments, but did not assess cerebral blood flow. A number of trials are currently underway to assess the effects of vasodilators on cerebrovascular function, including TREAT-SVDs, LACI-1/2^22^ and the OxHARP trial, the latter of which will specifically determine the effect of vasodilators on cerebral arterial pulsatility in patients with mild to moderate white matter hyperintensities.

Our study does have some limitations. Firstly, it was performed over a prolonged period in unselected patients in a follow-up clinic. It therefore included a large number of frail and elderly participants in whom physiological assessments could not always be performed and who are more prone to artefacts due to poor temporal bone windows. However, this population therefore also reflects the broad spectrum of patients most at risk of future events, and includes the more elderly and frail patients at the greatest risk of progression of small vessel disease. Secondly, in this study we have not reported the relationship with small vessel disease and clinical outcomes. However, this study was focused on the possible cause of increased cerebral pulsatility, which has previously been shown to strongly correlate with small vessel disease. With longer follow-up in this population, we will be able to determine the direct and indirect associations of each component of this pathway with clinical outcomes. However, ultimately an interventional study will be needed to prove the effect of changes in blood pressure or aortic pulsatility on cerebral haemodynamics and resulting clinical outcomes.

The relationships between arterial stiffness, aortic pulsatility, cerebrovascular pulsatility, small vessel disease and stroke risk, as well as potential associated effects of cerebrovascular reactivity and autoregulation, represents a constellation of physiological mechanisms that are potential targets to prevent progression of cerebral small vessel disease, recurrent stroke and cognitive decline. Our study clarifies the underlying mechanisms that can be targeted to reduce cerebral pulsatility, their relative importance and their comparative distribution within the population, with a key focus on aortic stiffness and pulsatility. As such, it provides a framework to understand the premorbid factors leading to the changes in this population, a baseline for ongoing assessment of risk and disease progression, and a series of therapeutic targets to test in clinical trials, both to determine their physiological effects and ultimately their effects on clinical outcomes.

## Conclusions

Cerebral arterial pulsatility is strongly associated with aortic pulsatility, which in turn depends upon aortic stiffness and augmentation of aortic pressure. This study has defined the relative distribution of these indices within the population, demonstrating potential for significant impact at the population level through treatments to reduce cerebral arterial pulsatility through targeting aortic and systemic arterial pulsatility.

## Supplemental Material

sj-pdf-1-jcb-10.1177_0271678X20969984 - Supplemental material for Physiological determinants of residual cerebral arterial pulsatility on best medical treatment after TIA or minor stroke Click here for additional data file. Supplemental material, sj-pdf-1-jcb-10.1177_0271678X20969984 for Physiological determinants of residual cerebral arterial pulsatility on best medical treatment after TIA or minor stroke by Alastair JS Webb, Amy Lawson, Linxin Li, Sara Mazzucco, Peter M Rothwell and for the Oxford Vascular Study Phenotyped Cohort in Journal of Cerebral Blood Flow & Metabolism
