# Supplementary material for: Physiological determinants of residual cerebral arterial pulsatility on best medical treatment after TIA or minor stroke
Source: J Cereb Blood Flow Metab. 2020 Nov 5;41(6):1463–71. doi: 10.1177/0271678X20969984 (PMC8138338; doi:10.1177/0271678X20969984)

SUPPLEMENTAL DATA

Physiological determinants of residual cerebral arterial pulsatility on best medical treatment after TIA or minor stroke

Alastair JS Webb, Amy Lawson, Linxin Li, Sara Mazzucco, Peter M Rothwell, for the Oxford Vascular Study Phenotyped Cohort

Supplemental Table 1. Distribution of demographic indices is shown for all patients, for patients undergoing either pulse wave analysis (PWA) or transcranial ultrasound and for patients undergoing both measures, with high quality recordings and not in AF. P-values are presented for patients not included in the analysis with patients with either PWV or TCD performed.

	All	Had either PWV Or TCD	
	Mean (SD)	Mean (SD)	p-val
	1013	981	
Age	67 (13.3)	66.9 (13.4)	<0.001
Sex	457 (45.1)	433 (44.1)	<0.001
Hypertension	782 (77.2)	761 (77.5)	0.11
Diabetes	119 (11.7)	112 (11.4)	0.07
Smoking			
- Current	164 (16.2)	158 (16.1)	0.69
- Ever	542 (53.5)	524 (53.4)	0.75
FHx of stroke	303 (29.9)	298 (30.3)	0.07
AF			
- During test	54 (5.3)	50 (5.1)	0.07
- Ever	74 (7.3)	69 (7)	0.07
Dyslipidaemia	819 (80.8)	791 (80.5)	0.33
Heart Failure	16 (1.6)	14 (1.4)	0.031
HTN med			
- CCB	458 (45.2)	444 (45.2)	0.87
- ACE	438 (43.2)	426 (43.4)	0.51
- ARB	133 (13.1)	128 (13)	0.67
- Diuretic	325 (32.1)	314 (32)	0.78
- Beta-blocker	150 (14.8)	147 (15)	0.38
Antiplatelet	857 (84.6)	832 (84.7)	0.30
Anticoagulant	87 (8.6)	82 (8.4)	0.15
Statin	792 (78.2)	764 (77.8)	0.19

Supplemental Table 2. Differences in indices between patients with or without specific demographic characteristics. Results are presented for univariate comparisons, and for estimated marginal means following adjustment for age, sex and cardiovascular risk factors.

	Aortic Measures								Cerebral Measures					
	PWV		SBP		DBP		PP		PSV		EDV		PI	
	No	Yes	No	Yes	No	Yes	No	Yes	No	Yes	No	Yes	No	Yes
Univariate														
**Female**	9.7	9.7	121.4	124.7	74.6	71.3	46.8	53.5	78.6	85.7	38.5	40.7	0.79	0.83
**Diabetes**	9.5	11.1	122.8	123.1	73.2	72.7	49.6	50.6	81.2	84.2	39.4	39.2	0.8	0.86
**Smoking**														
- **Ever**	9.9	9.6	123.1	122.6	73	73.3	50.1	49.5	82.2	81	39.6	39.2	0.81	0.81
- **Current**	9.8	9	122.9	122.7	72.8	74.6	50.1	47.9	81.2	83.1	38.9	41.6	0.82	0.75
**HTN**	8.8	10.6	118.8	126.8	72.6	73.8	46.4	53	82.7	80.3	41.7	36.9	0.75	0.87
**Dyslipidaemia**	9.0	9.9	120.8	123.4	74.2	72.9	46.6	50.5	82.8	81.2	41.2	39	0.78	0.81
Adjusted														
**Female**	10.5	10.3	123	125.8	74.5	71.4	48.3	54.5	80.7	88.1	38.2	40.8	0.83	0.87
**Diabetes**	9.8	11	125	123.8	73.7	72.2	51.1	51.7	82.1	86.7	39.1	39.8	0.82	0.88
**Smoking**														
- **Ever**	10.6	10.1	125.1	123.7	73.3	72.5	51.6	51.2	84.7	84.1	39.6	39.4	0.84	0.85
- **Current**	10.1	10.6	122	126.8	72.4	73.4	49.7	53.2	83.9	84.9	39.7	39.2	0.84	0.85
**HTN**	9.9	10.8	121.6	127.2	71.6	74.3	50.1	52.8	84.8	84	40.3	38.7	0.82	0.87
**Dyslipidaemia**	10.4	10.3	124.7	124.1	73.4	72.5	51.2	51.6	84.5	84.3	39.1	39.9	0.86	0.83

Supplemental Table 3. Associations with demographics variables. Results are presented for general linear models as univariate analyses, and following adjustment for age, sex and cardiovascular risk factors. Associations are reported as the unstandardized beta-coefficient and p-values.

	**Aortic Measures**						**Cerebral Measures**					
	**SBP**		**DBP**		**PP**		**PSV**		**EDV**		**PI**	
	**B**	**p**	**B**	**p**	**B**	**p**	**B**	**p**	**B**	**p**	**β**	**p**
Univariate												
**Age**	0.41	<0.001	-0.11	<0.001	0.48	<0.001	-0.17	0.001	-0.35	<0.001	0.008	<0.001
**Female Gender**	3.74	0.003	-2.95	<0.001	7.68	<0.001	7.08	<0.001	2.26	0.003	0.039	0.008
**Diabetes**	-0.004	1	-0.545	0.6	1.827	0.28	3.037	0.17	-0.177	0.88	0.061	0.009
**Smoking**												
- **Ever**	-0.95	0.45	0.11	0.87	-0.66	0.54	-1.22	0.38	-0.46	0.55	0.002	0.9
- **Current**	-0.74	0.67	1.95	0.03	-2.6	0.07	1.87	0.32	2.71	0.007	-0.07	<0.001
**HTN**	5.99	<0.001	0.07	0.93	5.49	<0.001	-5.08	0.002	-6.37	<0.001	0.11	<0.001
**MI**	-0.32	0.91	-3.64	0.012	1.87	0.44	1.34	0.68	-2.5	0.15	0.099	0.003
**Dyslipidaemia**	-0.62	0.64	-0.96	0.17	1.43	0.21	-1.93	0.2	-2.91	<0.001	0.06	<0.001
**Creatinine**	0.009	0.74	-0.012	0.43	0.012	0.63	-0.064	0.06	-0.086	<0.001	0.001	<0.001
**Weight**	-0.03	0.2	0.02	0.16	-0.04	0.014	-0.02	0.33	0.01	0.66	-0.001	0.028
**BMI**	-0.18	0.14	0.09	0.19	-0.25	0.022	-0.18	0.21	0.03	0.71	-0.003	0.02
Adjusted												
**Age**	0.41	<0.001	-0.11	<0.001	0.48	<0.001	-0.17	0.012	-0.35	<0.001	0.008	<0.001
**Female Gender**	3.74	0.007	-2.95	<0.001	7.68	<0.001	7.08	<0.001	2.26	<0.001	0.039	0.009
**Diabetes**	-0.004	0.95	-0.545	0.29	1.827	0.14	3.037	0.026	-0.177	0.56	0.061	0.001
**Smoking**												
- **Ever**	-0.95	0.33	0.11	0.34	-0.66	0.91	-1.22	0.7	-0.46	0.73	0.002	0.39
- **Current**	-0.74	0.015	1.95	0.3	-2.6	0.035	1.87	0.63	2.71	0.66	-0.07	0.72
**HTN**	5.99	0.09	0.07	0.09	5.49	0.95	-5.08	0.021	-6.37	<0.001	0.114	0.08
**MI**	-0.32	0.44	-3.64	0.003	1.87	0.85	1.34	0.16	-2.5	0.68	0.099	0.1
**Dyslipidaemia**	-0.62	0.08	-0.96	0.45	1.43	0.33	-1.93	0.86	-2.91	0.55	0.06	0.48
**Creatinine**	0.009	0.47	-0.012	0.15	0.012	0.73	-0.064	0.3	-0.086	0.6	0.001	0.26
**Weight**	-0.03	0.74	0.02	0.97	-0.04	0.79	-0.02	0.8	0.01	1	-0.001	0.53
**BMI**	-0.18	0.74	0.09	0.25	-0.25	0.23	-0.18	0.15	0.03	0.46	-0.003	0.18

Supplemental Table 4. Differences in key indices according to demographic characteristics, limited to patients with both PWA and TCD indices, not in AF. Results are presented for univariate comparisons, and for estimated marginal means following adjustment for age, gender and cardiovascular risk factors.

	Aortic Measures								Cerebral Measures					
	PWV		SBP		DBP		PP		PSV		EDV		PI	
	No	Yes	No	Yes	No	Yes	No	Yes	No	Yes	No	Yes	No	Yes
Univariate														
**Female**	9.7	9.4	119.1	122.7	74.7	71.2	44.5	51.7	79.4	86.4	38.5	41	0.8	0.83
**Diabetes**	9.4	11.3	120.7	120.6	73.3	72.5	47.4	48.2	82.1	84.4	39.6	39	0.81	0.88
**Smoking**														
- **Ever**	9.6	9.5	120.7	120.7	72.8	73.7	48	47.1	83.3	81.4	39.9	39.2	0.82	0.81
- **Current**	9.7	9	120.6	120.9	72.7	75.8	48	45.2	82.1	83.5	39.1	41.6	0.83	0.76
**HTN**	8.7	10.6	116.1	125.5	72.5	74	43.7	51.5	83.5	81.1	41.8	37.1	0.75	0.88
**Dyslipidaemia**	8.6	9.8	119.5	120.9	74.4	73	45.4	48	83.6	82	41.4	39.1	0.77	0.83
Adjusted														
**Female**	10.6	10.3	121.3	125.2	74.6	71.4	46.8	53.8	81.3	88.5	37.9	40.6	0.84	0.87
**Diabetes**	9.6	11.3	123.9	122.6	74	72.1	50	50.7	83	86.8	39.2	39.3	0.83	0.89
**Smoking**														
- **Ever**	10.6	10.3	123.5	122.9	73.2	72.8	50.5	50.2	85.6	84.2	39.5	39	0.86	0.86
- **Current**	10.1	10.7	120.6	125.9	72.1	73.9	48.6	52	84.3	85.5	39.5	39	0.85	0.87
**HTN**	10	10.8	119.6	126.8	71.6	74.4	48.2	52.5	85.3	84.5	40.1	38.4	0.83	0.89
**Dyslipidaemia**	10.4	10.4	124.7	121.8	73.4	72.7	51.5	49.1	84.9	84.8	38.7	39.8	0.87	0.84

Supplemental Table 5. Physiological determinants of cerebrovascular indices. Results of general linear models are presented for each cerebral blood flow index (PSV, EDV and PI) for model 1: including aortic pulse wave velocity (PWV), aortic systolic blood pressure (Aor-SBP) and Aortic diastolic blood pressure (Aor-DBP); or for model 2: including all factors in model 1 plus Aortic augmentation index (Aor-Aix) and R-R interval, the inverse of heart rate. Both models are presented unadjusted for clinical characteristics, adjusted for age and gender and adjusted for age, gender and cardiovascular risk factors (diabetes, history of hypertension, current smoking, ever smoking).

	**MCA Peak Velocity**						**MCA Trough Velocity**						**MCA Pulsatility Index**					
	**Physiology**		**Adj Age/Sex**		**Adj CV RFs**		**Physiology**		**Adj Age/Sex**		**Adj CV RFs**		**Physiology**		**Adj Age/Sex**		**Adj CV RFs**	
	**β**	**p**	**β**	**p**	**β**	**p**	**β**	**p**	**β**	**p**	**β**	**p**	**β**	**p**	**β**	**p**	**β**	**p**
Model 1	**R^2 =**	0.01	**R^2 =**	0.06	**R^2 =**	0.07	**R^2 =**	0.12	**R^2 =**	0.24	**R^2 =**	0.25	**R^2 =**	0.37	**R^2 =**	0.42	**R^2 =**	0.43
PWV	-0.08	0.054	0.06	0.221	0.05	0.389	-0.27	<0.001	-0.05	0.297	-0.03	0.559	0.31	<0.001	0.18	<0.001	0.12	0.003
Aor-SBP	0.1	0.028	0.11	0.027	0.12	0.018	-0.16	<0.001	-0.09	0.037	-0.06	0.223	0.41	<0.001	0.32	<0.001	0.29	<0.001
Aor-DBP	-0.11	0.014	-0.12	0.013	-0.12	0.018	0.18	<0.001	0.1	0.024	0.08	0.096	-0.46	<0.001	-0.36	<0.001	-0.34	<0.001
Model 2	**R^2 =**	0	**R^2 =**	0.05	**R^2 =**	0.07	**R^2 =**	0.12	**R^2 =**	0.22	**R^2 =**	0.23	**R^2 =**	0.41	**R^2 =**	0.46	**R^2 =**	0.49
PWV	-0.03	0.471	0.13	0.021	0.12	0.044	-0.26	<0.001	-0.04	0.444	-0.01	0.817	0.38	<0.001	0.25	<0.001	0.19	<0.001
Aor-SBP	0.02	0.668	0.03	0.57	0.04	0.558	-0.13	0.014	-0.09	0.086	-0.06	0.296	0.25	<0.001	0.2	<0.001	0.16	<0.001
Aor-DBP	-0.08	0.111	-0.09	0.116	-0.08	0.166	0.15	0.002	0.08	0.107	0.05	0.331	-0.4	<0.001	-0.32	<0.001	-0.28	<0.001
Aor-Aix	0.07	0.119	0.07	0.192	0.08	0.114	-0.02	0.581	0.02	0.627	0.04	0.416	0.13	<0.001	0.06	0.135	0.07	0.066
R-R int.	0.01	0.906	0.05	0.292	0.06	0.193	-0.09	0.039	-0.04	0.313	-0.05	0.289	0.13	<0.001	0.11	0.001	0.15	<0.001

Supplemental Figure 1: Distributions of cerebral physiological indices per quintile of aortic stiffness or the comparable aortic pressure measure. Results are presented as the mean and confidence interval of each index, split by gender. Results are presented for aortic systolic blood pressure (SBP), aortic diastolic blood pressure (DBP), aortic pulse pressure (PP), MCA peak systolic velocity (PSV), MCA end diastolic velocity (EDV) and MCA pulsatility index (PI).

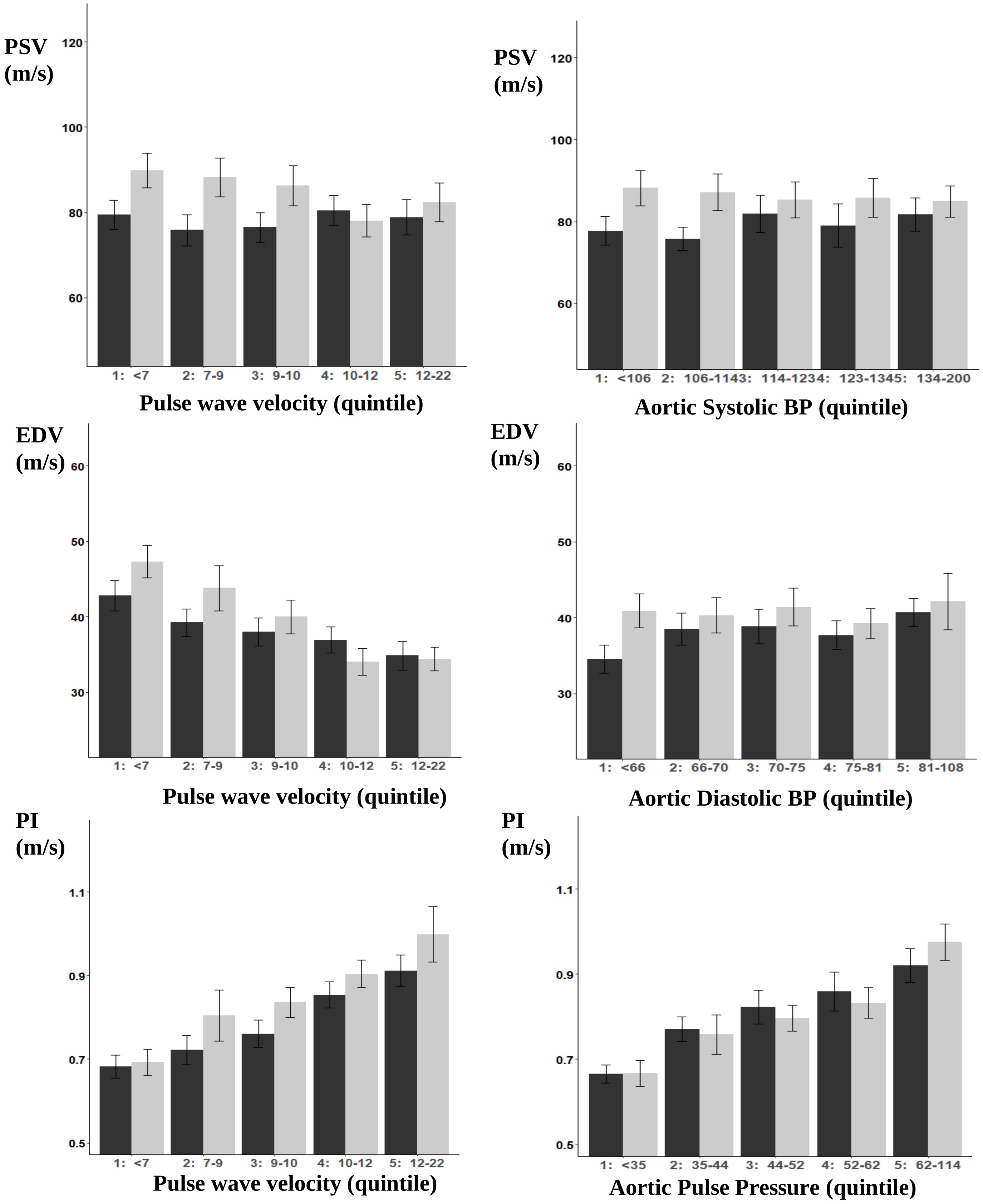

Supplement: sj-pdf-1-jcb-10.1177_0271678X20969984 - Supplemental material for Physiological determinants of residual cerebral arterial pulsatility on best medical treatment after TIA or minor stroke [file sj-pdf-1-jcb-10.1177_0271678X20969984.pdf]
